# Characterization of the IL-17 and CD4+ Th17 Cells in the Clinical Course of Dengue Virus Infections

**DOI:** 10.3390/v12121435

**Published:** 2020-12-13

**Authors:** Luis Alberto Sánchez-Vargas, Karina Guadalupe Hernández-Flores, Pablo Thomas-Dupont, Irma Yadira Izaguirre-Hernández, Elvis Efraín Sánchez-Marce, Ricardo Remes-Ruiz, Salvador Fonseca-Coronado, Pablo Augurio Hernández-Romano, María Estrella Flores-Collins, Héctor Vivanco-Cid

**Affiliations:** 1 Laboratorio Multidisciplinario en Ciencias Biomédicas, Instituto de Investigaciones Medico-Biológicas, Universidad Veracruzana, Veracruz 91700, Mexico; kimicoluis@hotmail.com (L.A.S.-V.); karinhernandez@uv.mx (K.G.H.-F.); lito_093@hotmail.com (P.T.-D.); yamadi17@hotmail.com (I.Y.I.-H.); 2 Hospital Regional de Alta Especialidad de Veracruz, Servicios de Salud de Veracruz, Veracruz 91700, Mexico; bono_marce@hotmail.com (E.E.S.-M.); riruiz@yahoo.com.mx (R.R.-R.); 3 Facultad de Estudios Superiores Cuautitlán, Universidad Nacional Autónoma de México, Cuautitlán-Izcalli 54740, Mexico; fonsecacoronado@yahoo.com.mx; 4 Laboratorio de Biología Molecular, Centro Estatal de la Transfusión Sanguínea (CETS), Boca del Rio, Veracruz 94294, Mexico; pabloahr@yahoo.com.mx; 5 Hospital General de Boca del Río, Servicios de Salud de Veracruz, Veracruz 94290, Mexico; estrellacollins@hotmail.com

**Keywords:** dengue, IL-17-producing cells, IL-17

## Abstract

The aims of this study were to determine the involvement of interleukin 17 (IL-17) and IL-17-producing cells in dengue pathogenesis. Blood samples from dengue virus (DENV)-infected patients were collected on different days after the onset of symptoms. Patients were classified according to 1997 World Health Organization guidelines. Our study examined 152 blood samples from dengue fever (DF, *n* = 109) and dengue hemorrhagic fever (DHF, *n* = 43) patients and 90 blood samples from healthy controls (HC). High serum concentrations of IL-17A and IL-22 were also associated with DHF (IL-17A [DHF vs. DF, *p* < 0.01; DHF vs. HC, *p* < 0.0001]; IL-22 [DHF vs. DF, *p* < 0.05; DHF vs. HC, *p* < 0.0001]). Moreover, there was a positive correlation between serum levels of IL-17A and IL-23, a key cytokine that promotes IL-17-based immune responses (r = 0.4089, *p* < 0.0001). Consistent with the IL-17-biased immune response in DHF patients, we performed ex vivo activation of peripheral blood mononuclear cells (PBMCs) from DHF patients and flow cytometry analysis showed a robust IL-17-biased immune response, characterized by a high frequency of CD4+IL-17+ producing cells. Our results suggests IL-17-producing cells and their related cytokines can play a prominent role in this viral disease.

## 1. Introduction

Dengue is the most prevalent mosquito-borne viral disease in tropical and subtropical areas. Worldwide, dengue virus (DENV) infects approximately 400 million people annually in more than 100 countries [1]. DENV belongs to the Flaviviridae family, which also includes other major human pathogens such as West Nile virus, Zika virus, Japanese encephalitis virus, yellow fever virus, Saint Louis encephalitis virus and Murray Valley encephalitis virus. Infection with one of the four closely related DENV serotypes can cause a wide spectrum of clinical features from asymptomatic disease, undifferentiated febrile illness, dengue fever (DF), dengue hemorrhagic fever (DHF), or dengue shock syndrome (DSS) [2]. Whereas a primary DENV infection provides lifelong serotype-specific protection, secondary infections with a distinct serotype (heterologous) present an increased risk for the development of severe disease symptoms [3,4].

Clinical outcomes of dengue can be influenced by several different factors, including viral factors such as viral load, serotype and virulence that are associated with different genotypes [5,6]. The genetic background of the host as well as polymorphisms in specific genes can also contribute to resistance or susceptibility to severe dengue clinical manifestations. Other important host factors in dengue pathogenesis include immune responses to the virus. Severe manifestations of DENV infections can be related to high levels of sub-neutralizing antibodies and activation of cross-reactive T cells, which produce a pro-inflammatory “cytokine storm” that produces vascular permeability that can lead to shock and death. Cytokines produced during DENV infections play a major role in either protection or susceptibility to the dengue severity. Severe DENV infections are characterized by elevated circulating levels of inflammatory cytokines and chemokines such as interferon gamma (IFN-γ), tumor necrosis factor alpha (TNF-α), interleukin (IL)-1β, IL-2, IL-4, IL-6, IL-7, IL-8, IL-18, macrophage chemo-attractant protein 1 (MCP-1), macrophage migration inhibitory factor (MIF) and granulocyte macrophage colony-stimulating factor (GM-CSF) [7,8,9,10]. A massive activation of adaptive immune cells, especially cross-reactive memory CD4+ T cells, has been reported to be a critical event in dengue hemorrhagic fever (DHF) pathogenesis [11,12]. Previous studies implicated Th1 and Th2 cell responses during the immune response to dengue wherein in vitro and ex vivo analyses of cytokine patterns in serum samples of patients with severe DHF showed a shift from a Th1 to Th2 response during the clinical course of the disease [13,14]. However, the studies described before are not conclusive and the role for other immune cell from innate and adaptive immunity and also other cytokine profiles in dengue infection remains unknown.

In the last two decades, the lineage of T helper 17 (Th17) cells has been one of the most widely studied cell populations and they are considered the major source of IL-17 cytokine production, an important pro-inflammatory mediator with multiple biological and pleiotropic effects. Th17 cells are characterized by producing interleukin 17 (IL-17A), IL-17F, IL-21, and IL-22 [15]. In response to certain stimulations, Th17 cells also produce TNF-α and IL-6. Th17 cells can be derived from naïve CD4+ T cells in response to TGF-β, IL-1β, IL-6 and IL-21 [15,16,17]. IL-23 has been found to be vital for Th17 cell maintenance [18]. Th17 cells are also involved in acute and chronic inflammatory diseases, mainly in the pathogenesis of autoimmune and allergic diseases [19]. Th17 cells and their effector cytokines also mediate host defensive mechanisms in response to different pathogens, particularly extracellular bacterial and fungal infections [20]. The involvement of Th17 cells has been well established in the immune response to human immunodeficiency virus (HIV) [21,22,23], hepatitis C virus (HCV) [24,25,26] and hepatitis B virus (HBV) [27,28], although the mechanism by which Th17 cells participate in antiviral defenses remains poorly understood. Moreover, to date the involvement of Th17 cells in medically important mosquito-borne flavivirus diseases is unclear. Other important IL-17-producing cells include CD8+ T cells, γδ T cells, invariant NKT (iNKT) cells and others thymus independent, such as group 3 innate lymphoid cells (ILCs).

The objectives of this study were to determine the frequency of CD4+IL-17-producing cells and the levels of related cytokines (IL-17A, IL-17F, IL-22, and IL-23) in dengue patients relative to healthy controls (HC), as well as, how these levels are affected in primary and secondary infections and related to the severity of infection (DF and DHF).

## 2. Material and Methods

### 2.1. Subjects

Dengue patients were recruited in the city of Veracruz, Mexico, and 152 blood samples from DF (*n* = 109) and DHF (*n* = 43) patients were tested. All serum samples were frozen at −80 °C until analysis. DF samples were collected from three urban health centers (“Anastasio Iturralde”, “21 de Abril”, and “El Coyol”) and DHF samples were collected from the Hospital Regional de Alta Especialidad de Veracruz during outbreaks that occurred between June 2012 and November 2014. During the study period, dengue virus serotype 2 and 4 circulated in Veracruz city [29].

A single blood sample was collected from each patient. Based on the time of clinical illness, we classified dengue patients into febril (1–3 days after disease onset), defervescence (4–7 days after disease onset) and convalescence (samples collected on days 8–10 after disease onset) and as having DF or DHF according to clinical and laboratory criteria of the World Health Organization 1997 (WHO 1997) [30]. DHF patients were clinically managed in the hospital for a median of 4 days. All DHF patients had laboratory evidence of thrombocytopenia (<100,000 counts/mm^3^) and a positive tourniquet test. Plasma leakage was estimated from multiple hematocrit determinations, and for some DHF cases pleural effusion was documented by chest *X*-ray. There were no fatal cases included in the present study. Children under 16 years old and patients older than 60 years old were excluded in order to make groups comparable by age. Pregnant women were also excluded from our study. To rule out the presence of other associated viral or bacterial infections, further serological tests for endemic pathogens such as leptospira, influenza A, hepatitis A and *Salmonella* spp. were carried out at the time of admission to the clinics.

Ninety serum samples from healthy controls (HC) were also tested. HC were enrolled in our study from the Centro Estatal de la Transfusión Sanguínea of Veracruz, which was in the same geographical region as the dengue patients. HC had no clinical signs or manifestations suggestive of dengue (no febrile symptoms) or other apparent illnesses in the previous 3 months. All HC were negative for Dengue laboratory tests and negative for all infectious disease screening assays performed in the blood bank.

### 2.2. Ethics Statement

Written consent to participate in the study was obtained from each participant after a full explanation of the study protocol and in accordance with the Ethics Committee of the Instituto de Investigaciones Medico-Biológicas, Universidad Veracruzana (Veracruz, México) guidelines. The institutional ethics review board of the Institute approved the study protocol.

### 2.3. Dengue Diagnosis

To confirm dengue diagnosis, samples were tested by reverse transcription–polymerase chain reaction (RT-PCR) and three reference anti-dengue enzyme-linked immunosorbent assays (ELISA): Platelia Dengue NS1 Ag Test (Bio-Rad Laboratories, Marnes-La-Coquette, France) and Dengue IgM (Panbio, Brisbane, Australia) and Dengue IgG (Panbio, Brisbane, Australia).

Alternatively to the ELISA kits, commercially available diagnostic tests SD BIOLINE Dengue Duo (Standard Diagnostic Inc., Gyeonggi-do, Korea) for the detection of NS1 antigen, IgM and IgG-dengue specific antibodies were used to analyze serum samples and the results were interpreted in accordance with the manufacturer’s instructions.

To discriminate among primary and secondary infections, a validated [31] commercial kit capture IgG ELISA (Panbio) was used. Primary dengue infection was defined for patients with positive results for RT-PCR, NS1 and/or IgM tests and negative IgG ELISA results. Secondary dengue infection was defined for IgG ELISA positive results (equivalent to a hemagglutination-inhibition titer of >2560) and at least one positive result for RT-PCR, NS1 and IgM results.

### 2.4. Measurement of Serum Cytokine Levels

Serum concentrations of IL-17 (A and F) and IL-23 were determined from dengue-positive patients and HC by a sandwich ELISA kit from eBioscience (San Diego, CA, USA). IL-22 was assessed using an ELISA kit from PeproTech, Inc. (Rocky Hill, NJ, USA), according to the manufacturer’s instructions. A microplate reader (Awareness Technology Inc., Palm City, FL, USA) was used to measure the optical density at 405 nm and 450 nm for the PeproTech ELISA and eBioscience kits, respectively, with a reference filter at 650 nm.

### 2.5. Flow Cytometry for CD4+IL-17 (Interleukin 17)-Producing Cells

Peripheral blood mononuclear cells (PBMC) were isolated from patients with secondary DENV infection. HC were age- and gender-matched to dengue-infected patients. PBMCs were isolated by density gradient using Lymphoprep^TM^ (Axis-Shield, Dundee, Scotland) according to the manufacturer’s instructions. PBMC were cryopreserved at 2 × 10^6^ cells/mL in RPMI-1640 media (Sigma-Aldrich, St. Louis, MO, USA) with 10% dimethyl sulfoxide (Sigma-Aldrich) and 40% fetal bovine serum (Biowest, Nuaillé, France) and kept at −160 °C until flow cytometry analysis was performed. After thawing, the viability of PBMC was more than 99%. PBMC were adjusted to a final concentration of 1 × 10^6^ cells/mL in RPMI 1640 medium (Sigma-Aldrich), supplemented with 10% inactivated fetal bovine serum (Biowest) and 1% Antibiotic/Antimitotic Solution 100× (Sigma-Aldrich). Because, all cytokine detection assays in lymphocyte cells are low-frequency analysis, PBMC were seeded in 24 well plates, and stimulated with 50 ng/mL PMA (Sigma-Aldrich) and 1 µg/mL ionomycin (Sigma-Aldrich) to stimulate the intracellular production of IL-17 in the presence of GolgiStopTM (BD Bioscience, San Jose, CA, USA) for 5 h. PMA/ionomycin stimulation was used according to the BD Pharmingen protocol for human Th17 stimulation and it is a widely used strategy for quantifying IL-17-producing cells and to evaluate intracellular cytokines from different Lymphocyte cell subpopulations, including Th17-derived cytokines [32,33,34,35,36]. Cells were stained for surface markers and intracellular cytokines according to the manufacturer’s protocol. Cells were incubated for 30 min at 4 °C with the following surface marker antibodies: anti-human CD3-FITC (fluorescein isothiocyanate) clone HIT3a and anti-human CD4-PE (phycoerythrin) clone SK3 from BioLegend (San Diego, CA, USA). Cells were washed twice with 0.9% saline solution (SS) and fixed with 4% paraformaldehyde for 20 min. Cells were then washed twice with SS and permeabilized with Perm/Wash Buffer (BD Bioscience) for 15 min and stained with anti-human IL-17A (Alexa Fluor 647) clone BL168 (BioLegend). Appropriate isotype controls were included in all experiments. Samples were analyzed using an Accuri C6 Flow cytometer (Becton-Dickinson, Franklin Lakes, NJ, USA). Compensation was carried out using commercial CompBeads (BD Biosciences) stained with individual fluorochromes. Flow cytometry data were analyzed using Accuri C6 software.

### 2.6. Statistical Analysis

Normality of data distribution was tested by the Kolmogorov–Smirnov test. The data were represented as medians with 25% and 75% interquartile ranges (IQR) or means with standard deviations (SD). Non-parametric Mann–Whitney U or Student’s *t*-tests were used to compare two independent groups as appropriate. Kruskal–Wallis (KW) or analysis of variance (ANOVA) tests were used to compare three or more independent groups where indicated. When the KW or ANOVA tests indicated a statistically significant difference, Dunn’s or Holm–Sidak multiple comparison tests, respectively, were performed to determine between which groups the differences were sustained. Correlation was evaluated using Spearman’s correlation test. GraphPad Prism v5.01 software was used to analyze the data. *p* values < 0.05 were considered statistically significant.

## 3. Results

### 3.1. Patient Characteristics

A total of 152 patients with dengue symptoms and without clinical evidence of other infectious diseases were enrolled in the study. The median age of the dengue patients was 26 (range: 12–66 years old). The gender ratio was 1:2.6 (male:female). From the 152 dengue-positive patients, 109 (71.7%) and 43 (28.3%) were classified as having DF and DHF, respectively, according to WHO 1997 clinical criteria [30]. Patients were also grouped according to illness phase (febrile, defervescence and convalescence) according to the same WHO 1997 clinical criteria. DF and DHF patients had similar median age and gender percentage distributions. Days of illness and laboratory findings are summarized in Table 1. As expected, hematocrit and lymphocyte counts were higher in DHF patients relative to DF patients, while the platelet count was lower in DHF compared to DF samples (*p* < 0.05). Using the criteria described in the Methods section, of the 152 patients, 52 were confirmed to have a primary infection and 100 patients were confirmed as having secondary DENV infections. No patients with hemorrhagic manifestations who were included in this study fulfilled WHO criteria for DSS.

### 3.2. Elevated Serum Levels of IL-17A, IL-17F, IL-22 and IL-23 in Dengue Patients

**Figure 1:**
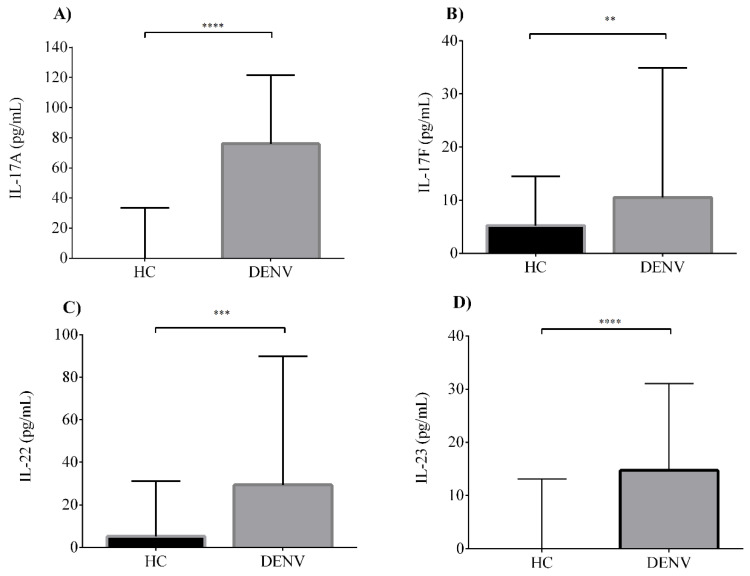
Elevated serum levels of interleukin 17A (IL-17A), IL-17F, IL-22 and IL-23 are produced in dengue infections. Serum levels of (**A**) IL-17A, (**B**) IL-17F, (**C**) IL-22 and (**D**) IL-23 in healthy controls (HC) (*n* = 78) compared with dengue virus (DENV)-infected patients (*n* = 128). Individual data are shown and the horizontal line is the median value. Statistical difference is based on the Mann–Whitney test. ** *p* < 0.01, *** *p* < 0.001, **** *p* < 0.0001.

Little is known about the dynamics of IL-17-related cytokine production in the context of dengue pathogenesis. Furthermore, there is limited and contradictory scientific evidence for the potential role of IL-17A in dengue infection severity. Our group reported before the involvement of a Th17-related cytokine, IL-21, in the clinical course of DENV infections [37]. However, the dynamics of production and biological role for other related cytokines such as IL-17F, IL-22 and IL-23 have not been described. To clarify the involvement of all these related cytokine members in dengue infection pathogenesis, we performed a serum analysis for the IL-17 signature cytokines IL-17A, IL-17F, IL-22 and IL-23. We first compared the serum concentrations between dengue-infected patients and HC (Figure 1). Dengue patients showed elevated serum levels for IL-17A (median 76.0 pg/mL, IQR 24.6–121.4), IL-17F (median 10.5 pg/mL, IQR 2.1–34.9), IL-22 (median 29.3 pg/mL, IQR 0–89.8) and IL-23 (median 14.7 pg/mL, IQR 6.2–31.0) compared to HC (IL-17A, median 0 pg/mL, IQR 0–33.5; IL-17F, median 5.2 pg/mL, IQR 1.1–14.5; IL-22, median 5.2 pg/mL, IQR 0–31.2; and IL-23, median 0 pg/mL, IQR 0–13.1). Between dengue patients and the HC, there were statistically significant differences for all IL-17-related cytokines tested (*p* < 0.01).

### 3.3. Dynamics of Production of IL-17-Related Cytokines at Different Phases of Illness

**Figure 2:**
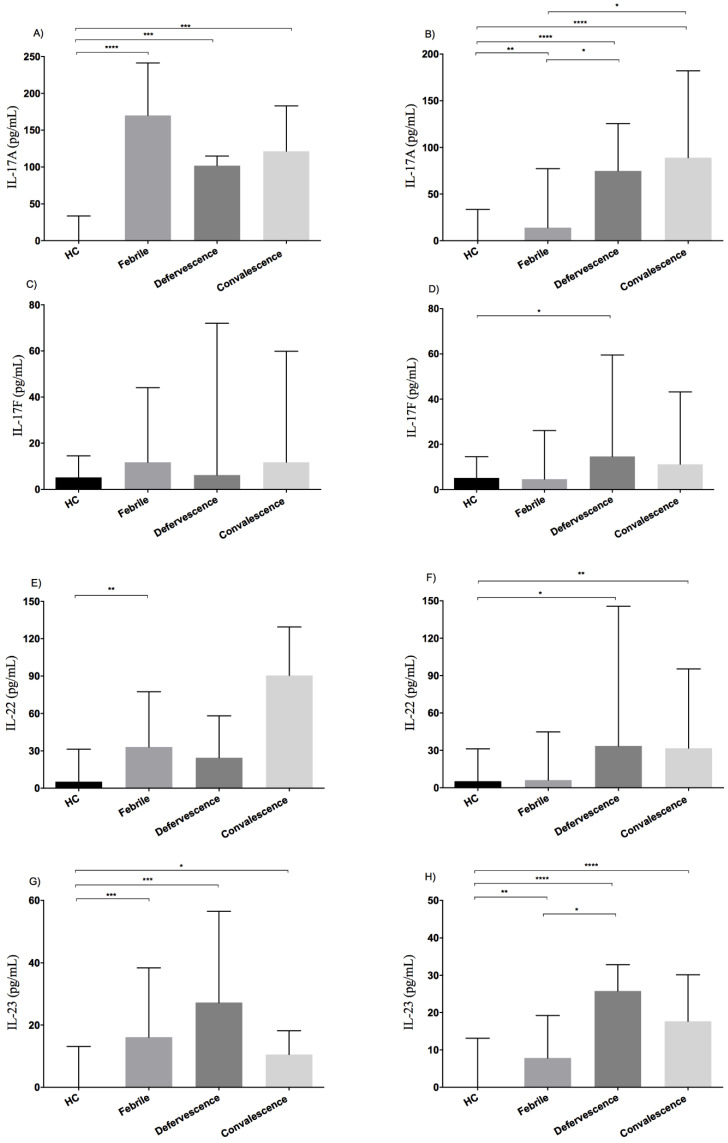
Dynamics of production of IL-17-related cytokines at different illness phases. Dengue patients were grouped according to illness phase (febrile, defervescence and convalescence) and infection type (primary and secondary). Median values of IL-17A serum levels are shown for primary (**A**) and secondary dengue infections (**B**), for IL-17F in primary (**C**) and secondary (**D**) infections, for IL-22 in primary (**E**) and secondary (**F**) infections and for IL-23 in primary (**G**) or secondary (**H**) infections. Individual data are shown and the horizontal line is the median value. Statistical difference is based on the Kruskal–Wallis with Dunn’s multiple comparison post-tests. * *p* < 0.05, ** *p* < 0.01, *** *p* < 0.001, **** *p* < 0.0001.

To perform a detailed analysis of IL-17-related cytokine production during the clinical course of dengue infections, serum concentrations in dengue patient samples were grouped according to illness phase (febrile, defervescence and convalescence) and infection type (primary vs. secondary). In primary infections, there was no differential expression among the IL-17-related cytokines in febrile, defervescence and convalescent illness phases (*p* > 0.05) (Figure 2A–D). Yet when we compared the serum levels of IL-17-related cytokines in patients to HC, we found that IL-17A and IL-23 were differentially expressed at different illness phases relative to HC (*p* < 0.05) (Figure 2A,D). In addition, there was an increase in IL-22 serum levels in the febrile phase of primary dengue patients relative to HC (Figure 2C), (*p* = 0.009). Significantly, serum levels for IL-17A and IL-22 are detectable even during the very early infection period (days 2 and 4) of primary infections compared to HC, suggesting that innate immune cell sources may exist that allow the production of these cytokines during the early stages of primary dengue infections. In contrast, patients with secondary infections showed a different expression pattern for the IL-17-related cytokine profile during the clinical course of secondary DENV infection (Figure 2E–H). IL-17A and IL-23 levels showed differential expression between patients who were in the febrile, defervescence and convalescence phases (*p* < 0.05), with the highest serum levels for all IL-17-related cytokines during secondary infections observed in the defervescence and convalescence phases (Figure 2E–H).

### 3.4. Serum Levels of IL-17A and IL-22 Are Associated with Dengue Infection Severity

**Figure 3:**
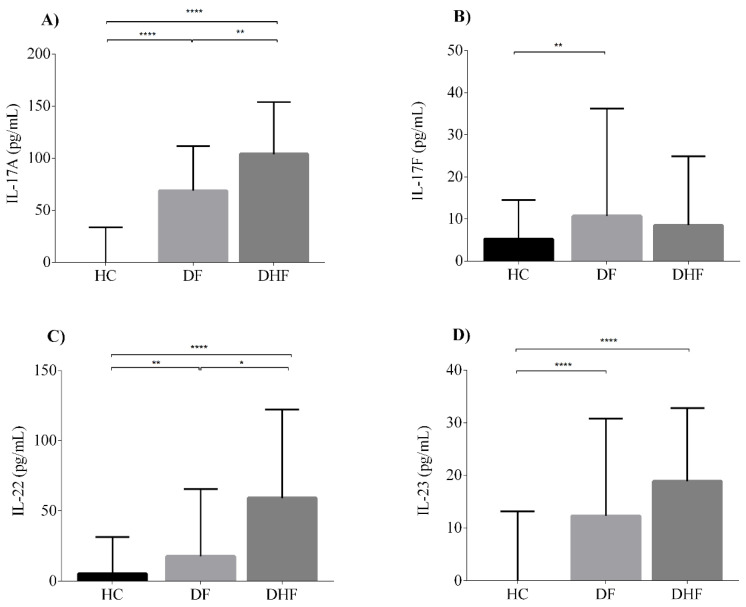
IL-17A and IL-22 are associated with DHF severity. Serum levels of (**A**) IL-17A, (**B**) IL-17F, (**C**) IL-22 and (**D**) IL-23 in healthy controls (HC) (*n* = 78) compared with dengue fever (DF, *n* = 97) and dengue hemorrhagic fever (DHF, *n* = 31). Individual data are shown and the horizontal line is the median value. Statistical difference is based on the Kruskal–Wallis with Dunn’s multiple comparison post-tests. * *p* <, 0.05 ** *p* < 0.01, **** *p* < 0.0001.

To investigate the potential association between high levels of IL-17-related cytokines and dengue severity, we next classified the patients into two groups, DF or DHF, based on clinical and laboratory and clinical criteria stated in the WHO 1997 guidelines [30]. Interestingly, serum concentrations of IL-17A and IL-22 were significantly higher in DHF patients (IL-17A median 103.8 pg/mL, IQR 69.5–153.8; IL-22 median 59.0 pg/mL, IQR 23.4–122.1) than in DF patients (IL-17A median 68.7 pg/mL, IQR 12.6–111.7; IL-22 median 17.6 pg/mL, IQR 0–65.4) (*p* < 0.05) (Figure 3). IL-17F and IL-23 displayed relatively similar serum levels in both clinical forms with no statistically significant differences (IL-17F, DF median 10.7 pg/mL, IQR 2.8–36.2 vs. DHF median 8.5 pg/mL, IQR 0–24.9; IL-23, DF median 12.3 pg/mL, IQR 5–30.8 vs. DHF median 18.9 pg/mL, IQR 9.1–32.8) (Figure 3).

### 3.5. High Serum Levels of IL-17A and IL-23 Are Correlated during Dengue Infections

**Figure 4:**
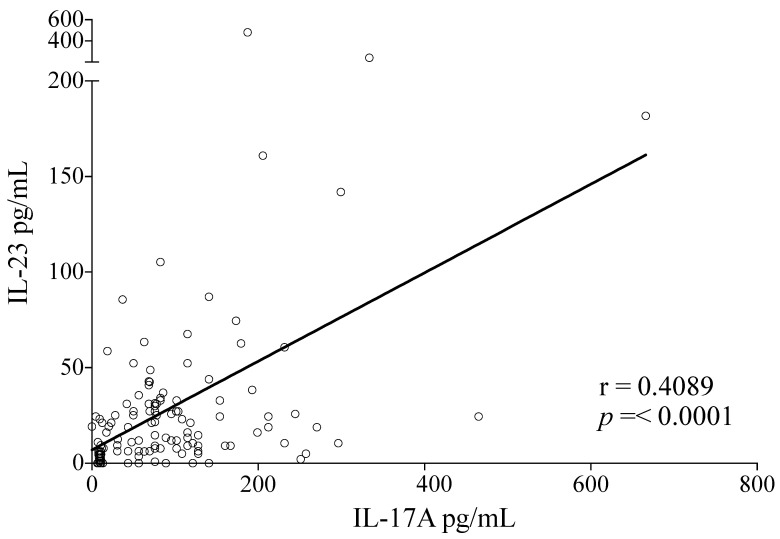
Serum levels of IL-17A and IL-23 are correlated during the clinical course of dengue infection. A positive correlation was found between IL-17A and IL-23 levels (r = 0.4089, *p* = < 0.0001) in patients with DENV infection (*n* = 128). Statistical difference is based on Spearman’s correlation test.

IL-23 is a vital cytokine for the maintenance of Th17 cells and production of IL-17. The role and dynamics of production for IL-23 during DENV infections are unknown, so we analyzed whether there was an association between production of IL-17A and IL-23 in DENV infected patients. We found that there was indeed a positive correlation between serum IL-17A and IL-23 levels in DENV-infected patients (r = 0.4089, *p* < 0.0001) (Figure 4) confirming a promoting role of IL-23 to the IL-17-biased immune response in DENV infections.

### 3.6. CD4+ IL-17-Producing Cells in DENV-Infected Patients Are Associated with Disease Severity

**Figure 5:**
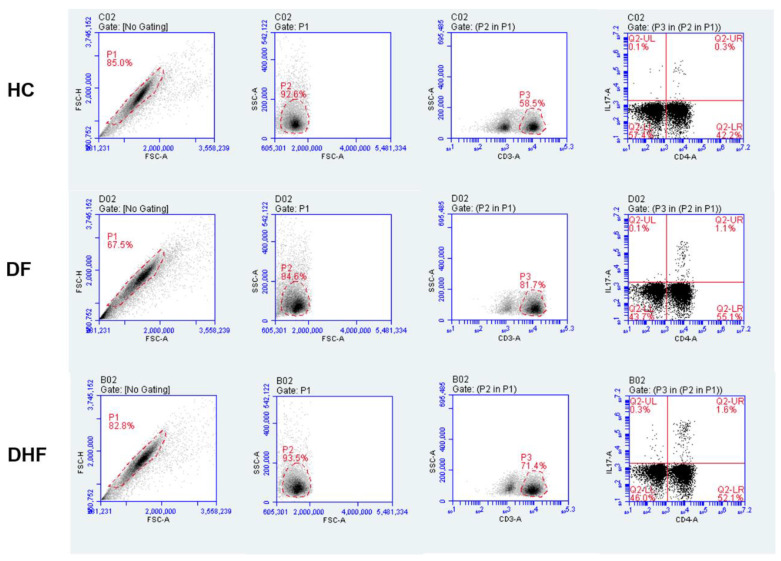
Strategy for IL-17-producing cell analysis. Dot plots are representative of one HC, DF and DHF patient. Ten thousand events were acquired from lymphocyte regions based on CD3-FITC staining. Percentages of IL-17-producing cells were identified in the CD4+ and CD4- region with the quadrants established based on isotype controls. CD3+ CD4+ IL-17+ cells were considered to be Th17 cells mainly.

**Figure 6:**
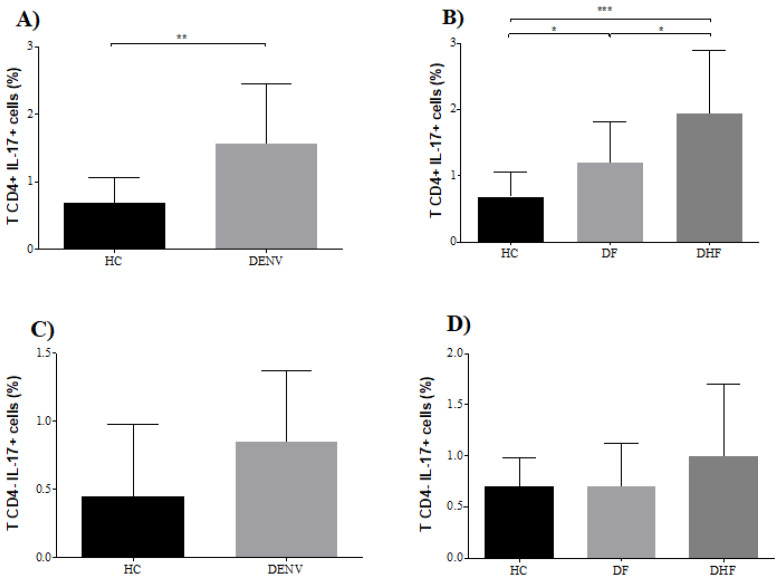
Higher percentage of CD3+CD4+IL-17-producing cells in DENV-infected patients. (**A**) Percentage of IL-17+ cells in HC (*n* = 12) and DENV-infected patients (*n* = 24). (**B**) Percentage of IL-17+ cells in HC (*n* = 12), DF patients (*n* = 12) and DHF patients (*n* = 12). (**C**) Percentage of CD3+CD4-IL-17+ cells in HC (*n* = 12) and DENV-infected patients (*n* = 24). (**D**) Percentage of CD3+CD4-IL-17+ cells in HC (*n* = 12), DF patients (*n* = 12) and DHF patients (*n* = 12). Statistical difference is based on Student’s *t*-test (**A**), Mann–Whitney test (**B**), one way analysis of variance (ANOVA) with Holm–Sidak’s multiple comparison test (**C**) and the Kruskal–Wallis with Dunn’s multiple comparison post-tests (**D**). * *p* < 0.05, ** *p* < 0.01, *** *p* < 0.001.

Given that our previous results suggested that elevated serum levels of IL-17A are associated with disease severity during DENV infections, we next analyzed a subgroup of 24 patients with secondary dengue infection for the potential contribution of CD4+ IL-17-producing cells to the pathogenesis. The selected patients were in the critical phase of the illness (i.e., defervescence) when serum IL-17A levels peaked. We compared the percentages of CD4+IL-17-producing cells in 12 secondary DF cases and 12 secondary DHF patients, and then compared the results against those for 12 age- and gender-matched HC. The gating strategy for the flow cytometry analysis of IL-17-producing cells is shown in Figure 5. Patients with DENV infection showed a higher percentage of CD4+ IL-17-producing cells (mean 1.6%, SD ± 0.87) than HC (mean 0.7%, SD ± 0.38) (*p* < 0.01) after polyclonal activation (Figure 6A). When we analyzed the patients according to clinical severity, we found that there were significant percentages of CD4+IL-17-producing cells in DHF patients (mean 1.9%, SD ± 0.9) compared to DF patients (mean 1.2%, SD ± 0.6) (*p* < 0.05) (Figure 6B).

In contrast, no differences were found in the percentage of CD4-IL-17-producing cells, either in DENV-infected patients relative to HC (*p* = 0.2192), or among clinical severities (*p* = 0.2952) (Figure 6C,D).

## 4. Discussion

Dengue is a medically important mosquito-borne viral disease. However, despite recent progress in the understanding of dengue physiopathology, many issues remain to be addressed. One unresolved issue for this complex viral disease is the role that cellular immunity plays in dengue pathogenesis. Here, we demonstrated the involvement of IL-17-producing cells, during immunopathogenesis of dengue infection. Although IL-17-producing cells and their effector cytokines are known to have both pathological and protective roles during different inflammatory conditions, their role and dynamics in flavivirus-associated diseases remains poorly understood.

A major observation from our study is the higher percentage of IL-17-producing cells (CD3+CD4+ T cells) in DENV-infected patients and how the increased percentage of this cell subset were associated with the severity of secondary dengue infections. Interestingly, we observed no differences in the percentage of CD3+CD4-IL-17+ T cells, either in DENV-infected patients relative to HC or in relation to clinical severity, suggesting that CD8+ T cells are not a major source of IL-17A in human dengue infections.

At this point, there are two major limitations to our study. First, our investigations of immunological markers on IL-17-producing cells was not exhaustive and did not include specific phenotypic markers of TH17 cells such as CD161, CCR6, or lineage-specific transcription factors (ROR-gammat) but focused on two well-described markers of surface (CD4 and CD3) for T helper cells and the cytokine expression pattern: IL-17A, a well studied lineage-specific intracellular marker for Th17 cells. Secondly, at the time of patient recruitment and samples analysis, it was not possible perform an antigen specific activation using DENV or purified dengue antigens. Nevertheless, a higher frequency of CD4+IL-17- producing cells was clearly demonstrated after polyclonal activation of PBMCs from DHF patients compared with DF patients and HC. In addition, we show that high serum levels of IL-17A, IL-17F, IL-22 and IL-23, which are hallmark cytokines that are associated mainly with Th17 cells, are produced during human dengue infections. Few studies have examined the role of IL-17 in dengue pathogenesis. Our results for the association between IL-17A levels and dengue severity are consistent with recent scientific reports. In a study involving 38 Venezuelan infants infected with DENV, Duran et al. [38] found that IL-17 serum levels were highest in severe dengue cases relative to patients with dengue without warning signs (DNWS). Furthermore, in a 211-patient cohort Jain et al. [39] observed a tendency towards elevated serum levels of IL-17A in severe cases relative to non-severe cases, although the differences were not statistically significant. Furuta et al. showed in a prospective study performed with 103 Vietnamese patients that the highest plasma concentrations of IL-17 are detected in DHF and DSS patients relative to DF patients and HC [40]. In a mouse model of dengue infection, IL-17 receptor-deficient mice infected with DENV-2 showed reduced lethality compared to wild-type (WT) mice, and treatment of DENV-2 infected mice with anti-IL-17 antibodies was associated with reduced disease severity [41]. These results, together with those from our study, strongly suggest that IL-17A production is correlated with dengue severity infections.

Regulation of homologous members of the IL-17 protein family such as IL-17A and IL-17F during dengue infections differed in our study. Whereas in secondary cases IL-17A was associated with dengue severity, there was no such association for IL-17F. Meanwhile, in primary infections we found similar serum levels for both IL-17 isoforms. Although in humans IL-17F and IL-17A are encoded by genes that are located in the same chromosomal region (6p12) [42,43,44] there is evidence for differences in gene regulation and expression of both isoforms in the context of infectious diseases [45]. IL-17A and IL-17F share 50% sequence homology and are likely produced as homodimers (IL-17A or IL-F) or heterodimers (IL-17A/IL-17F) [46]. In our study, we measured only homodimeric protein forms. Both IL-17 isoforms have overlapping biological functions, but the biological activity of IL-17A seems to be more potent than that of IL-17F [46].

The high levels of both IL-17 isoforms (IL-17A and IL-17F) observed in the early stage of primary dengue infections suggest that there are other relevant cell sources for IL-17 from innate immunity that occurs in the absence of a specific T cell response. In addition to Th17 cells, other cell types, including NKT cells, NK cells, γδ-T cells, ILCs, macrophages and neutrophils can produce IL-17 [47,48,49,50]. Thus, further studies with dengue-infected patients or in vitro infection studies with dengue virus are needed to explore the role of these other cells in dengue infection.

It is interesting to note that IL-17 serum levels were found to be higher in the primary febrile phase than in the febrile phase of secondary infections. Although the precise reason for these results is unclear, the average time of sample collection, the infecting serotype in patients with primary or secondary dengue infections and the pre-existing memory adaptive immune response in secondary dengue infections may modulate the production of IL-17.

The biological relevance of IL-17-producing cells and their involvement in viral pathogenesis has been studied for other viral infections such as HIV, HCV and HBV. HIV infections are associated with a low percentage of circulating Th17 cells [21,22,23]. Furthermore, IL-17 levels positively correlate with the percentage of Th17 cells, and both Th17 cells and IL-17 have a negative correlation with HIV plasma viral load [21]. The number of Th17 cells normalizes in HIV patients receiving effective antiretroviral treatments, which suggests that these cells could participate in the control of HIV infection [21].

Specific Th17 cell responses are reportedly induced in HCV-infected patients [24]. Patients with chronic HCV infection have increased proportions of both circulating and liver-infiltrating Th17 cells, which have a positive correlation with the severity of liver inflammation [25]. Moreover, high levels of IL-17 occur in HCV-infected patients compared to healthy donors, although no correlation with the viremic state was found [25,26]. In HBV infections, there is an increase in the frequency of Th17 cells in peripheral blood from chronic-infected HBV patients. In addition, a positive correlation between the frequency of Th17 cells and serum alanine aminotransferase (ALT) levels was found [27]. In another study, infiltrating Th17 cells were observed in the liver of HBV patients, and the frequency of Th17 cells was associated with viral load, ALT, and hepatitis activity index, suggesting these cells could play a role in exacerbating the disease [28].

A recent study has shown that CD4+ and CD8+ T cells isolated during the acute phase of coronavirus disease 2019 (COVID-19) patients produce tumor necrosis factor (TNF), interferon-γ, interleukin (IL)-2 and high levels of IL-17 after polyclonal activation, suggesting a detrimental role of IL-17 during the excessive inflammatory response observed in COVID-19 infection [51].

In the context of dengue infections, Th17 cells and other IL-17-producing cells may also play an important dual role during the immune response to DENV. First, the sustained IL-17A production during the clinical phases of primary infections is quite surprising. The biological role of IL-17A in primary DENV infections could be very different that in secondary infections.

Our findings in secondary infections support the hypothesis that IL-17-producing cells can play a detrimental role in disease immunopathogenesis by enhancing the inflammatory response during the clinical course of secondary dengue infections. The increased production of IL-17A, in a synergic effect with other inflammatory mediators, could in turn result in severe dengue pathology during secondary infections. Both in vitro and in vivo results have shown that IL-17A induces a monocyte-macrophage lineage to produce pro-inflammatory cytokines such as IL-1β, IL-6 and TNF-α, thus amplifying inflammatory responses [52,53,54]. Given the central role of human monocytes as an inflammatory cell population and natural host cell for dengue virus replication, the biological effects of IL-17 on dengue-infected monocytes could have important biological implications with respect to worsening of both viral infection and systemic inflammation.

Moreover, IL-17A has been reported to mediate neutrophil recruitment, activation, and migration [55]. In this regard, IL-17 could play an important role in dengue infections by activating neutrophils and inducing the release of inflammatory mediators from these cells. Indeed, IL-17A is a powerful inducer of IL-8 in various cell populations such as macrophages, epithelial cells, smooth muscle cells and synovial fibroblasts [52,56,57,58,59,60]. High levels of IL-8 were previously shown to occur in patients with dengue virus infection and correlate with neutrophil degranulation as well as with some clinical and hemodynamic variables [61]. Therefore, the in vivo biological effects of IL-17A on neutrophils should be explored in future studies.

Other potential detrimental effects of high IL-17A levels that mainly occur during secondary DHF cases could be the activation of endothelial cells that in turn induces the expression of adhesion molecules, production of inflammatory mediators and vascular permeability. IL-17A was reported to induce endothelial cell activation in an extracellular signal-regulated kinase (ERK) signaling-dependent pathway [62]. Interestingly, IL-17A induces the production of von Willebrand factor (VWF) by endothelial cells. Severe dengue cases have been associated with exocytosis of Weibel–Palade bodies as well as increased circulating levels of VWF that may contribute to thrombocytopenia and complications in severe dengue cases [63]. A key biological effect of IL-17 on endothelial cells is the induction of apoptosis through caspase-3 and -9 activation and up-regulation of the Bax/Bcl-2 ratio [64]. Endothelial cell apoptosis is induced by different mechanisms, including direct viral infection [65,66] and by the effect of pro-apoptotic cytokines, including IL-17 [64].

Other pathogenic effects of IL-17A on endothelial dysfunction include the induction of the gene expression of chemokines and pro-inflammatory mediators, including IL-8, CCL20, IL-6 and IL-15. IL-17 induces also the expression of metalloprotease genes such as matrix metalloproteinase (MMP)-2 and MMP-9 [67,68]. Notably, high circulating levels of MMP-9 have been associated with dengue severity [69]. IL-17A was recently demonstrated to be a novel predictor of vascular dysfunction in rheumatoid arthritis [70], and elevated plasma levels of IL-17A are associated with cardiovascular risk [71]. Taken together, this evidence supports the hypothesis that IL-17 production during dengue infections could act in a synergistic mechanism with other pro-inflammatory mediators to produce detrimental effects by promoting the activation and damage of the vascular endothelium in DHF cases.

Other pathogenic roles for Th17 cells that involve IL-17A production in dengue infections could be associated with the inhibition of the cytotoxic activity of both NK cells and CD8+ T cells. Th17 cells inhibit T cell cytotoxicity in a mouse model of persistent viral infection mediated by Theiler’s encephalomyelitis virus [72]. In vitro, IL-17A inhibits CD8+ T cell–mediated apoptosis on target infected cells through a mechanism that may involve inhibition of CD8+ T cell cytotoxic function by IL-17-induced blockage of the Fas–FasL pathway [72]. In vivo, IL-17A also reduces NK cell activity in vaccinia virus-infected mice, while in vitro IL-17A inhibits the expression of cytotoxic effector molecules such as granzyme B, perforin and IFN-γ produced by splenic NK cells [73].

Our study also provides initial evidence concerning the involvement and production of IL-22 during the clinical course of dengue infection. In a mouse model of dengue, IL-22^-/-^ deficient mice were more susceptible to DENV infection than WT mice, suggesting a protective role for this cytokine [41]. Treatment of dengue-infected IL-22 deficient mice with anti-IL-17 antibodies reverts to the disease phenotype, confirming an antagonist function for both cytokines in this model of dengue infection [41]. In our study, we found for the first time that DENV infection in humans is associated with higher IL-22 serum levels in DHF patients relative to DF patients, which suggests an important role for IL-22 in terms of regulating the inflammatory effects of IL-17 during dengue infection. Given that IL-22 receptor expression is restricted to cells of non-hematopoietic origin, the biological relevance of IL-22 in severe dengue infections is quite intriguing. In a mouse model, overexpression of IL-22 modulates factors involved in coagulation, including fibrinogen levels and platelet numbers, and cellular constituents of blood, such as neutrophil and red blood cells (RBC) counts [74]. Based on these studies, which demonstrate hepatoprotective and hepatoproliferative roles for IL-22 [75,76,77,78], we hypothesize that IL-22 could have a potential protective role in modulating inflammation in certain target organs such as the liver and gut, which are two tissues that are affected during severe dengue infections.

Finally, since IL-23 is a vital cytokine for the maintenance of Th17 cells and regulation of IL-17 production, we demonstrated the dynamics of IL-23 production in dengue infections. IL-23 is mainly produced by dendritic cells and macrophages, which both act as permissive cells for dengue infection [79,80,81]. Previous studies in lymphocytes demonstrated that IL-23 induces STAT3 phosphorylation that is essential for maintaining the Th17 cell profile [82]. The positive correlation of IL-23 and IL-17 serum concentrations suggests that IL-23 is produced in vivo to support a Th17 profile during dengue infections.

We previously reported the dynamics of the production of IL-21, another important Th17-related cytokine, during the clinical course of DENV infections. High serum levels of IL-21 are produced mainly in the convalescent phase of primary infections and are associated with the production of DENV-specific IgM and IgG antibodies. [37]

In summary, many detrimental (IL-17A, IL-17F) or protective effects (IL-21, IL-22) can be inferred from the biological activities of these related cytokines. IL-17A produced mainly by Th17 cells during secondary infections could play a relevant role during the “cytokine storm” that arises during an aberrant and uncontrolled immune response to dengue infection and participates in a synergistic mechanism with other pro-inflammatory cytokines to amplify systemic inflammation, which together suggest that Th17 and other IL-17-producing cells can play key roles in the immunopathogenesis of dengue infection.

## Figures and Tables

**Table 1:** Demographic and clinical characteristics of the study patients with dengue fever (DF) and dengue hemorrhagic fever (DHF).

Characteristics	DF *n* = 109 (71.7%)	DHF *n* = 43 (28.3%)	*p* Value
Male sex *n*= (%)	38 (34.9)	20 (46.5)	0.1986
Days of illness median (range)	5 (1–28)	7 (2–12)	<0.0001
Age (years) median (range)	26 (12–66)	26 (12–63)	0.8585
Hematocrit % mean (SD)	39.2 (3.9)	41.3 (4.8)	0.0147
Platelet (10^3^/mm^3^) median (range)	151.5 (23–465)	66 (16–246)	<0.0001
Lymphocytes (10^3^/mm^3^) median (range)	0.9 (0.2–4.8)	1.7 (0.4–6.1)	<0.0025
